# Exact exchange-correlation potential of an ionic Hubbard model with a free surface

**DOI:** 10.1038/srep02172

**Published:** 2013-07-10

**Authors:** V. Brosco, Z.-J. Ying, J. Lorenzana

**Affiliations:** 1 Istituto dei Sistemi Complessi CNR and Università di Roma “La Sapienza”, P. le A. Moro 2, I-00185 Rome, Italy

## Abstract

In Kohn-Sham density functional theory (DFT) the interacting electron problem is mapped into a noninteracting problem in an effective potential *v_KS_*. It is known that the charge gap of the interacting system is different from the gap of the effective problem due to a jump Δ*_xc_* in *v_KS_* when an electron is added but its magnitude and its role in the ubiquitous discrepancy between the experimental gaps and approximate DFT computations is poorly understood. Here we compute the exact *v_KS_* of a strongly interacting one-dimensional lattice model which can be driven from an ionic to a Mott insulating state. Presence of a “vacuum” region allows to determine the absolute value of *v_KS_*. We show that in the ionic regime Δ*_xc_* is determined by nearest-neighbor interaction, while in the Mott regime Δ*_xc_* is determined by on-site Hubbard interaction.

Density functional theory (DFT)^1^,^2^,^3^ plays a major role in our understanding of ground state properties of materials. However most approximate DFT approaches fail to predict the fundamental gap Δ*_C_* of insulators and semiconductors (band gap problem)^4^,^5^,^6^,^7^,^8^,^9^,^10^,^11^,^12^,^13^,^14^,^15^,^16^,^17^,^18^,^19^,^20^ in systems ranging from bulk Silicon^8^ to ZnO^13^ and other correlated insulators^14^. Here Δ*_C_* ≡ *I^N^* − *A^N^* where *I^N^* and *A^N^* indicate respectively the ionization energy and the electron affinity of the *N*-particle system, 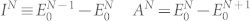 with 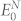 denoting the ground state energy of the *N*-particle system.

Almost all DFT computations are based on Kohn and Sham (KS) scheme^2^, in which the ground-state density *ρ* of *N* interacting electrons in an external potential is reproduced by a system of non-interacting electrons in an effective potential 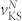. The effective potential can be expressed as the sum of three contributions: the external potential, *v*, the Hartree potential 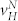, and a term which accounts for exchange and correlation effects, 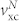. The latter is the functional derivative of a universal “divine functional”^21^ of the density whose precise form is not known.

As first discussed by Perdew *et al.*^4^,^5^ and by Sham and Schlüter^6^, even for the exact functional, the charge gap of an interacting system does not coincide with the single particle gap in the Kohn-Sham non-interacting system but there is a correction due to a discontinuity in the functional derivative 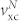 vs. the particle number, 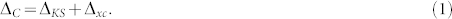
More precisely Sham and Schlüter^6^ consider a large periodic system and argue that the correction is given by 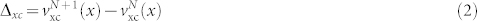
with the right hand side becoming a constant in the thermodynamic limit, independent of position *x* in the solid. Perdew et al. obtain a similar result by considering instead a finite open system^4^,^5^.

It is believed that the jump in the exchange correlation potential, which is absent in local and semi-local approximate functionals^10^,^13^,^20^, may account for the error on the fundamental gap^5^,^6^. The size of this effect is however controversial^7^,^8^,^9^,^10^,^11^,^12^,^13^,^14^,^15^,^16^,^17^,^18^,^19^,^20^ and even its existence^16^ has been questioned.

This debate, along with the need to understand and correct the deficiencies of approximate DFT approaches, has over the years concurred to focus the attention of the scientific community on two classes of systems: small systems (zero dimensional), whose exchange-correlation potential can be calculated exactly or very accurately^17^,^18^,^22^,^23^,^24^,^25^,^26^,^27^,^28^, and lattice models, where DFT schemes can be tested and analyzed in a controlled environment retaining many of the subtleties of the many-body problem^29^,^30^,^31^,^32^,^33^,^34^,^35^,^36^,^37^,^38^,^39^,^40^,^41^,^42^.

In a pioneering work Gunnarsson and Schönhammer^7^ studied a model of a one-dimensional spinless insulator and they found that Δ*_xc_* is small in the band insulating regime. Other authors have, however, argued that the discontinuity should be large and it should account for a large part of the band gap problem^8^,^9^,^11^. More recently Sagvolden and Perdew studied analytically and numerically the case of an hydrogen ion described by a statistical mixture so that the average number of particles can be fractional and showed rigorously the validity of the assumptions that lead to Eq. (1) in the special case where the particle number crosses *N* = 1. A rigorous investigation of these issues in an open many-particle system is however still lacking.

In this work we fill this gap using lattice DFT to investigate the band-gap problem. We calculate numerically the exact exchange-correlation potential of a correlated insulator described by a generalized Hubbard model which can be tuned continuously from an ionic to a Mott insulating regime^43^. Differently from previous works, we consider a Hubbard chain connected to a free surface. As we show below, this allows us to compute each term on the left and right of Eq. (1) separately enabling us to check the validity of the assumptions that lead to this equation and to clarify the physics in a context very different from previous studies. Indeed differently from Refs. 17,18,25,26,27,28 we consider a lattice model (rather than the continuum) and a many (rather than few) particle system and we go beyond Ref. 31 both by including a nearest-neighbor interaction and by using an exact reverse-engineered exchange-correlation potential. Following Sham and Schlüter^6^ the discontinuity Δ*_xc_* is estimated using finite differences and errors due to the finite size of our system are discussed.

We find that the contribution of Δ*_xc_* to the charge gap is non-negligible both in the ionic and Mott insulating regimes, and we are able to trace back this contribution to different interaction terms in the Hamiltonian giving simple analytical estimates. Eventually we study the structure of the exchange-correlation potential in the vacuum sites where we find a barrier close to the bulk-vacuum interface, which appears as the system enters the Mott phase, an effect similar to the one studied in Refs. 17,18,26 in the case of few particle systems and Ref. 25 in the case of an extended system but neglecting correlation effects.

Dealing with the physics of the Hubbard model with the language and the tools of DFT this work lies at the border between two fields. For this reason and to unify the language, our presentation emphasizes some aspects, which are known in one community but not always recognized in the other, in a somehow pedagogical way. In addition we provide a generalization of some celebrated results for DFT in the continuum to the lattice like Koopmans theorem and the relation between charge decay rate and ionization energy.

## Results

### Model

**Figure 1:**
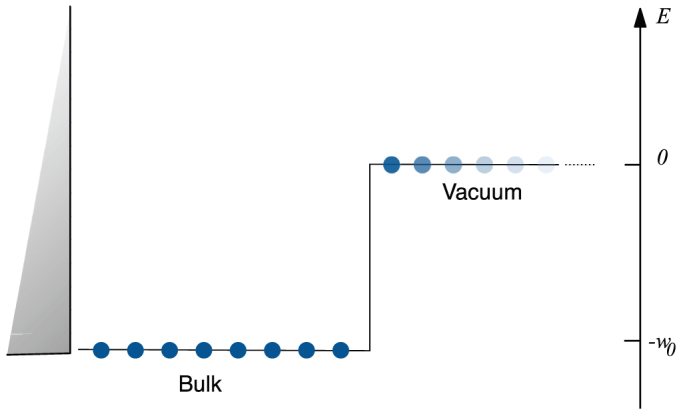
Model. Structure of the system consisting of *L_B_* bulk and *L_V_* vacuum sites. The bulk sites have a large binding energy, *w*_0_.

We consider a Hubbard chain of *L_B_* sites with a large binding energy, called “the bulk”, followed by a chain of *L_V_* sites with zero binding energy, termed “the vacuum”, with open boundary conditions as shown in Fig. 1. The bulk is thus a truly open system, *i.e.* the number of particle in the bulk is not fixed, this is crucial to completely determine the exchange-correlation potential.

The total Hamiltonian can be written as *H* = *T* + *H_U_* + *H_v_* with 
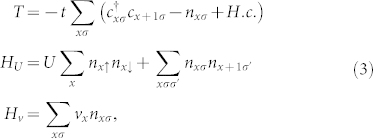
where 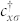 creates an electron with spin *σ* = ↑,↓ at site *x*, *U* and *V* are respectively the Hubbard interaction and nearest-neighbour interaction, *t* is the nearest-neighbour hopping and we set 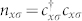. We included a constant energy shift in the lattice kinetic energy *T* so that single particle energies are measured from the bottom of the band.

In order to simulate the work function of a solid the potential in the bulk is taken as *v_x_* = −*w*_0_ + *δ*(−1)*^x^* where *w*_0_ is a large positive constant such that all particles in the system are bound in the bulk region and the second term is a site dependent potential. The potential in the vacuum is by definition *v_x_* = 0.

We apply DFT to this problem by considering the site occupancy *ρ_x_* = Σ*_σ_*〈*n_xσ_*〉 as the fundamental variable^7^,^29^. The density and the energy of the ground state are obtained using Lanczos exact diagonalization^44^. The exchange correlation potential is obtained from the exact density inverting the Kohn-Sham problem as discussed in Section Methods.

An important result of DFT, which we will use, is Koopmans' theorem^4^ which states that the highest occupied eigenvalue of the Kohn-Sham potential coincides with the ionization energy of the system. Notice that the usual uniform Hubbard model consisting of *T* + *H_U_* and periodic boundary conditions is particle-hole symmetric while the theorem distinguish between occupied and unoccupied states. It will become clear below that vacuum levels, which obviously break particle-hole symmetry, can not be neglected from the model in order to make the system compliant with the theorem.

### Homogeneous Hubbard bulk

We first consider the case of constant potential in the bulk (*δ* = 0) and vanishing nearest-neighbour interaction *V* = 0. This corresponds to the uniform Hubbard model which has been discussed in the framework of lattice DFT in Refs. 7,29,31,32,33.

**Figure 2:**
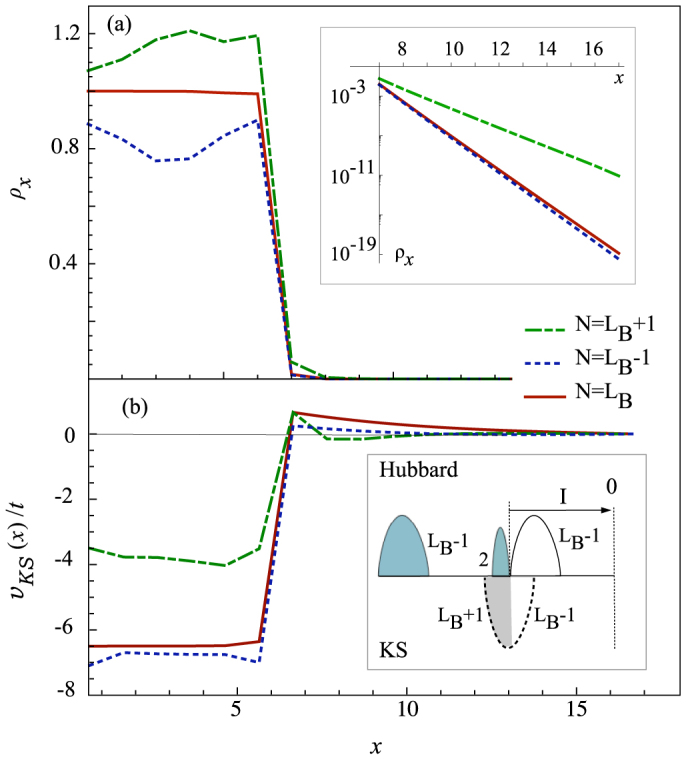
From the exact density to the exact KS potential. Panel (a) and (b) show respectively the charge density and the KS potential for *U* = 6*t* at three different fillings, namely *N* = *L_B_*, *N* = *L_B_* ± 1. The inset of panel (a) presents a logarithmic plot of the density in the vacuum while the inset of panel (b) presents a schematic comparison between the spectrum of the Hubbard model and of the effective KS system for *N* = *L_B_* + 1 which we use below to qualitatively explain the peak in the main panel (b) appearing for this filling close to the surface. Other parameters are: *w*_0_ = 8*t*, *δ* = 0, *L_B_* = 6 and *L_V_* = 11.

In the upper and lower panels of Fig. 2, we plot respectively the electron density and the exact effective potential for *U* = 6*t* and *w*_0_ = 8*t*. We consider in particular the case when the bulk is half-filled, *i.e. N* = *L_B_*, and the cases of a bulk above and below half-filling, *N* = *L_B_* ± 1.

As shown in the lower panel, while the change in the bulk effective potential in going from *N* = *L_B_* − 1 case to the *N* = *L_B_* is small and can be attributed to a O(1/*N*) effect, there is a sizable O(1) jump in going from *N* = *L_B_* to *N* = *L_B_* + 1. For all other fillings different from *N* = *L_B_* we find that the jump due to the addition of one particle is O(1/*N*).

Notice that the jump is confined to the region of bound charges and even there is not perfectly constant due to the finite-size of our system. Similar finite-size effects were discussed in Refs. 17,18,26 where the exchange-correlation potential of small systems in the continuum was calculated. These computations have shown that the jump due to the addition of a small but finite fractional charge 0 < *f* < 1 is spatially constant inside a region of radius *R* around the bound charges of the system with *R* → ∞ when *f* → 0^+^. Analogously we expect that in our case the jump remains constant within a region with a boundary defined by *R* → ∞ when *N* → ∞ keeping *N*/*L_B_* constant so that the excess density 1/*L_B_* → 0^+^. Such a limit is outside the reach of our numerical capabilities. Notice that here we are dealing with a many-body system with many electrons showing band behaviour while in Refs. 17,18,26, where the *f* → 0^+^ limit could be studied numerically, only one and two electron systems were considered. In support of our expectation, similar effects were observed by Horowitz *et al.* in the exact *exchange* potential of a jellium slab (c.f. Fig. 7 of Ref. 25) without the use of an ensemble density but partially populating a band. At first sight, since Horowitz *et al.* neglect correlation, it may appear that their effect is different from ours. However it is easy to see that it is enough that the functional has a discontinuity in the Kohn-Sham potential, no matter form what origin, to obtain this effect.

In order to obtain an estimate of the exchange-correlation contribution to the charge-gap for our finite system we follow Ref. 6 and use, 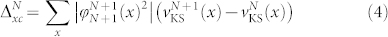
with *N* = *L_B_*. This equation can be seen as a weighted average of the jump where only the regions where the density of the less bound electron is finite contribute. Also the replacement of *v_xc_* by the whole KS potential makes no difference in the thermodynamic limit since only the exchange correlation part of the potential has a jump of order one. Equation (4) arises naturally when one computes the charge gap directly from the functional as it is done in Ref. 6 for the continuum and it is shown for the lattice case in the Supplementary material. There we also show how errors due to the finite difference estimate of the jump cancel to the lowest order in 1/*N*, giving on the whole a surprisingly small contribution even for relatively short bulk chains.

**Figure 3:**
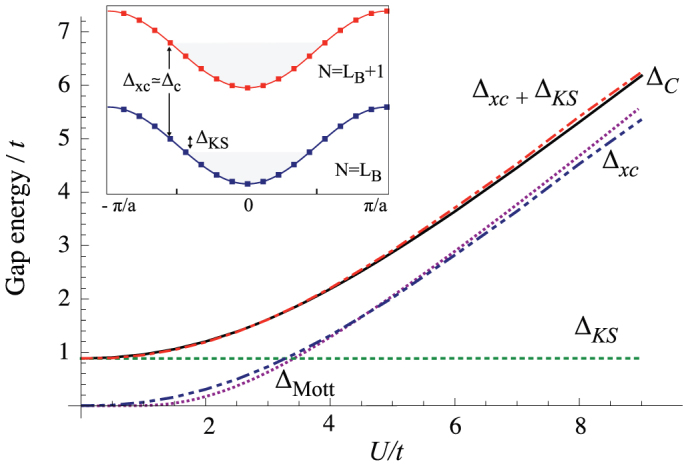
Mott gap and *xc*-potential jump. Exact charge gap Δ*_C_*, Kohn-Sham gap, Δ*_KS_* and contribution of the *xc*-potential jump, Δ*_xc_* for a half-filled Hubbard chain with a free surface as a function of the on-site Coulomb repulsion. Other parameters are as in Fig. 2. Δ*_Mott_* is the Mott gap for an infinite system calculated using Bethe Ansatz^47^. The inset shows Kohn-Sham band structure of a *uniform* Hubbard chain (periodic boundary conditions) at half-filling (*N* = *L_B_*) and with one added electron (*N* = *L_B_* + 1).

Fig. 3 shows the *U* dependence of the exact charge gap for the *N* = *L_B_* electron system with the ionization energy and the electron affinity energies obtained with the Lanczos computation. We also show Δ*_xc_* + Δ*_KS_*, where Δ*_KS_* is the exact KS gap, *i.e.* the gap in the spectrum of the effective non-interacting *N*-particle Kohn-Sham system.

We see that indeed Eq. (1) is well fulfilled. As discussed below the Kohn-Sham gap should vanish in the thermodynamic limit for a Hubbard chain so its finiteness is a finite size effect.

The charge density in the vacuum remains for all fillings much smaller than 1 and it decays exponentially as shown by the logarithmic plot in the inset of Fig. 2 (upper panel). The change in the density decay rate in the vacuum as the filling becomes larger than one (*N* > *L_B_*), reflects a change in the ionization energy. Indeed, as first discussed in Ref. 45, the density decay rate, *κ*, far from the surface of a bulk metal or a molecule is related to the ionization energy as 
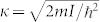
. For lattice systems we find in a similar way that the following relation holds: *κ* = 2 arccosh (1 + *I^N^*/2*t*). Therefore an accurate computation of the density profile in the vacuum region fixes the ionization energy and it allows to compute the absolute value of the Kohn-Sham potential in the bulk following the route outlined by Almbladh and von Barth in Ref. 46. There they provide a proof of Koopmans theorem of DFT^4^ relating the highest occupied KS eigenvalue to the ionization energy (see the Supplementary Material for a discussion of this theorem in the lattice and a proof of the above expression for *κ*).

The inset of Fig. 3 shows schematically the behaviour of Kohn-Sham bands in a large Hubbard chain which can be solved exactly with periodic boundary conditions^47^. Since the charge is uniform, Kohn-Sham potential is a constant which, without the vacuum, remains undetermined, *i.e.* any constant potential yields the correct ground state density. However we know from the exact solution that the chemical potential as a function of the filling has a jump at half-filling equal to the Mott-Hubbard gap Δ*_Mott_*. Then, if we loosely consider the atoms of the Hubbard chain to have a large constant binding energy *v_x_* = −*w*_0_ and to be immersed in a “vacuum” with zero binding energy, we expect the ionization energy to have a jump at half-filling due to the jump in the chemical potential. At the same time, by DFT Koopmans theorem, we expect Kohn-Sham potential and Kohn-Sham bands to shift rigidly so that Δ*_xc_* = Δ_*Mott*_, as shown schematically in the inset of Fig. 3. Indeed in Fig. 3 we also see that in spite of the bulk chain being short (*L_B_* = 6), Δ*_xc_* approximately coincides with Δ*_Mott_* for the infinite system calculated by Bethe Ansatz showing that this picture^7^,^31^ is correct and finite size corrections to Δ*_xc_* due to the use of Eq. (4) are negligible.

In Figure 2 we eventually note the appearance of a peak at the boundary between vacuum and bulk on the vacuum side with width and height depending on the filling. This peak has the same origin as the plateaus, appearing in computations of the exchange-correlation potential of finite systems^17^,^18^,^26^ and of jellium slabs^25^ with a small but finite amount of fractional charge *f*. Such behaviour was qualitatively understood in terms of the different ionization potential of the different components of the density^18^ and a similar explanation can be put forward here. Just as the leading (slowest) decay rate of the wave function is determined by the first ionization energy, ionization from deeper states will determine subleading decays rates which are important at short distances from the surface^46^. To better understand the origin of this peak it is thus useful to compare the photoemission spectrum of the Hubbard model to the corresponding Kohn-Sham spectrum. As an example, the inset of Fig. 2 displays a very schematic picture of the removal spectra of the two systems at large *U* above half-filling (shaded regions). In both cases the total weight is proportional to the number of occupied states, *N* = *L_B_* + 1, however in the Hubbard model only two occupied states, are available^48^ close to the chemical potential (binding energy −*I*), all the other states have much lower energy (binding energy ~ −*I* − *U*). On the contrary in the Kohn-Sham spectrum a full metallic band consisting *L_B_* + 1 states is available at binding energy ~ −*I* (the vacuum level corresponds to zero energy). It is not difficult to show that this large spectral difference implies different subleading decay rates, with a tendency of the Kohn-Sham system to have a charge density larger than that in the interacting system close to the boundary. This excess charge is eliminated by the appearance of the peak in the Kohn-Sham potential. Thus, the anomalous transfer of spectral weight in the Hubbard model, which is the hallmark of strong electron correlation^48^, reflects in the appearance of the peak. We remark that, although related, this peak does not have the same origin as the well-known correlation barrier studied in the context of dissociation of diatomic molecules and the step structure that appears in the case of heteronuclear molecules (see *e.g.* Refs. 27,28,49). Indeed while the barrier in the dissociation problem appears around the middle point between two atoms in a stretched molecule, the present one accounts for the short range behavior of the charge close to a bulk system. To understand this point it may be useful to consider the dissociation of a long chain into two identical fragments. In this case we expect that the Kohn-Sham potential will display both effects, two peaks or plateaus close to each fragment correcting the short-range decay of the density and a third one in the middle of the stretched bond.

### Charge gap in ionic and Mott-like insulators

We now come to the discussion of the transition between a Mott insulator and an ionic insulator. In order to simulate a binary compound we use the model introduced above with *δ* ≠ 0, a Hubbard interaction *U* equal on all atoms and a nearest neighbour repulsion *V*. The system shows a transition from an ionic insulating regime to a Mott insulating regime when *U* ~ 2*δ* + *zV* with *z* = 2 the coordination number^43^. In the atomic limit one finds that 
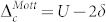
 in the Mott regime and 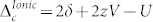 in the ionic regime with both gaps coinciding at the transition. Notice that the latter is larger than the nearest-neighbour charge transfer energy corresponding to the excitation of a Frenkel exciton 
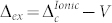
: this becomes relevant below when we discuss the jump in the exchange correlation potential.

**Figure 4:**
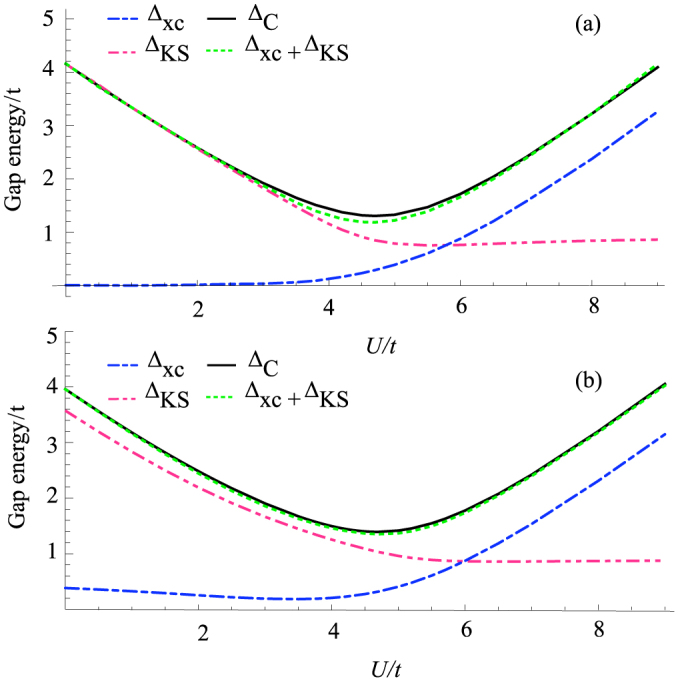
Contributions to the charge gap in the different regimes. Panel (a) and (b) show the different contributions to the gap, Δ*_KS_* and Δ*_xc_* and compare their sum to the exact charge gap calculated by Lanczos diagonalization, Δ*_C_*. The parameters in the two panels are chosen to have the same total charge gap at *U* = 0 in the small hopping limit. Parameters are in panel (a) *δ* = 2*t*, *V* = 0, in panel (b) *δ* = *t*, *V* = 0.5*t*. In both panels we set *w*_0_ = −6*t* + *U*/2 and the potential of the site closer to the bulk-vacuum boundary has been shifted to minimize boundary effects.

Figure 4 shows again that that Eq. (1) is well satisfied with negligible finite size corrections (see Supplementary Material for an explicit analytical expression of finite size corrections to the charge gap). Panel (a) and (b) show respectively the results for *V* = 0 and *δ* = 2*t*, and for *V* = 0.5*t* and *δ* = *t*. As one can easily check the total charge gap at *U* = 0 for small *t* is the same in the two cases. However in the first case we have 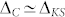 in the ionic insulator (small *U*) and 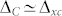 in the Mott-insulating phase (large *U*), while in the second case we have a finite contribution of Δ*_xc_* to the gap in both regimes. Clearly the appearance of a finite Δ*_xc_* in the ionic regime is linked to the presence of the non-local interaction *V*. This can be easily understood by considering the limit of weak tunneling *t* ≪ Δ*_ex_*. Using perturbation theory one easily finds that the amount of charge transferred from odd to even sites in the exact many-body solution is 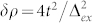. Neglecting surface effects, by symmetry, the difference in the Kohn-Sham potential between even and odd sites is equal to the Kohn-Sham gap. Applying the same perturbative argument to the Kohn-Sham system we arrive to the conclusion that to match the exact density Δ*_KS_* = Δ*_ex_*. Therefore to leading order 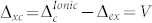. It is easy to check that these relations are valid in any dimension. They are in good agreement with the numerical results of Fig. 4 in the ionic regime for finite *t*.

## Discussion

We have computed the exact exchange correlation potential of a correlated extended system including the (usually undetermined) absolute value with respect to a vacuum level. This allowed to verify the assumptions leading to Eq. (1) in a context quite different to what has been previously done^18^. Namely an extended strongly correlated system on the lattice. In addition we have found that a surface correlation barrier appears in the effective potential of a correlated system when the removal spectrum of the system is very different from the removal spectrum of the Kohn-Sham system, as expected to occur in electron-doped Mott insulators.

For Mott insulators we have shown that the discontinuity of the exchange correlation potential equals the total charge-gap and it is of the order *U* for strong correlation, for a homogeneous Hubbard model we recover the results of Refs. 29,31. On the other hand in a strong ionic insulator we showed that the discontinuity is determined by the nearest-neighbour repulsion *V* which provides a simple estimate of this elusive quantity.

In general we expect that in strongly ionic insulators, to a good approximation, the Kohn-Sham gap matches the first Frenkel exciton and that Δ*_xc_* is given by its binding energy respect to the fundamental gap. While in ionic salts the Frenkel exciton is easily accessible experimentally, the fundamental gap is difficult to measure and is often obtained by a theoretical fit to the observed optical spectra^50^. In any case matching of the Frenkel gap by Δ*_KS_* puts a strong constraint on density functionals in strong ionic insulators.

## Methods

To calculate the exact Kohn-Sham (KS) potential, *i.e.* the effective non-interacting potential which corresponds to the exact density, we adopt the following strategy: we first obtain the ground-state density by applying Lanczos diagonalization^44^ to the “bulk + vacuum” chain, we then extract the KS potential by minimizing the difference between the KS density and the exact one for all values of the KS potential.

The KS density is as usual expressed in terms of KS orbitals, *ϕ_i_*(*x*, *σ*), as 
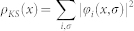


The orbitals *ϕ_i_*(*x*, *σ*) are in turn defined through the well-known KS equations, 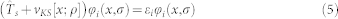
where 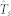 is defined by 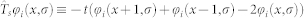, *v_KS_*[*x*; *ρ*] is the effective KS potential and *ε_i_* are the KS energies. To find the exact KS potential, we thus simply minimize the relative mean square error on the density *i.e.* we calculate: 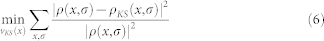
where *ρ* denotes the exact density obtained by Lanczos diagonalization. After the minimization the relative error on the density is smaller than 10^−5^ *i.e.* 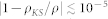. Such a high accuracy is necessary to correctly describe the asymptotic decay of the density in the vacuum and to satisfy “Koopmans theorem” of DFT^4^. This theorem, which identifies the highest occupied KS eigenvalue with the exact ionization energy, has been discussed by several authors for DFT in the “continuum”^4^,^46^,^51^ and has been also subject of controversies^52^,^53^,^54^,^55^. In the Supplementary Material we extend its validity to lattice systems.

## Author Contributions

All authors, V.B., Z.Y. and J.L., equally contributed to designing the research, understanding the numerical outcomes and reviewing the manuscript.

## Supplementary Material

Supplementary Information Supplementary material to Exact exchange-correlation potential of an ionic Hubbard model with a free surface
