# Supplementary material for: Exact exchange-correlation potential of an ionic Hubbard model with a free surface
Source: Sci Rep. 2013 Jul 10;3:2172. doi: 10.1038/srep02172 (PMC3707039; doi:10.1038/srep02172)
Supplement: Supplementary Information — Supplementary material to Exact exchange-correlation potential of an ionic Hubbard model with a free surface [file srep02172-s1.pdf]

Supplementary Material to "Exact exchange-correlation potential of an ionic Hubbard model with a free surface"

V. Brosco, Z.-J. Ying, J. Lorenzana[1]

[1]*ISC-CNR and Dipartimento di Fisica, University of Rome "La Sapienza", P.le A. Moro 2, I-00185 Rome, Italy*

(Dated: April 27, 2013)

Proof of Koopmans theorem in the lattice

The behavior of the density in the vacuum far from the bulk-vacuum border is determined by the bulk ionization energy and, in the Kohn-Sham picture, by the last occupied KS eigenvalue. This result is at the basis of the so-called "Koopmans theorem of DFT" and, as discussed in the article, it connects the offset between the bulk and vacuum KS potentials to the long-range decay of the density in the vacuum. Here we prove Koopmans theorem for lattice DFT.

To prove "Koopmans theorem" in the lattice we follow the route outlined by Almbladh and von Barth[1] and we introduce the quasiparticle amplitudes, $f_s(x) = \langle N-1, s|c_{x\sigma}|N, 0\rangle$, where $|N, 0\rangle$ and $|N, s\rangle$ denote respectively the ground and the s-th excited state of the full N-electron Hamiltonian.

Considering that in the vacuum sites the density is very small and decays exponentially with the distance from the surface for our parameter choice then, following the same reasoning as in Ref. 1, one can show that many-body effects become asymptotically irrelevant. Therefore, deep in the vacuum, the quasiparticle amplitudes satisfy the Schrödinger like equation

$$-t(f^s_{x+1,\sigma} + f^s_{x-1,\sigma} - 2f^s_{x,\sigma}) - f^s_{x,\sigma}\Delta E_s = 0 \tag{1}$$

where ΔE_s indicates the difference between the ground state energy of the N-particle system and the energy of the s-th excited level of the $N-1$-particle system, $\Delta E_s = E^N_0 - E^{N-1}_s$. Solving this recursive equation we obtain the following result for the decay of the ground-state quasi-particle amplitude, f_0 in the vacuum:

$$f_0(x) \simeq e^{-\beta x} \tag{2}$$

with $\beta = \text{arccosh}(1 + \frac{I_N}{2t})$, I_N being the ionization energy $I_N = -\Delta E_0$ of the N-particle system. For comparison in the continuum the decay rate is given by $f_0(r) \propto e^{-\kappa r}$ with $\kappa = \sqrt{2m_e I}$[2] where m_e denotes the electron mass.

Now considering that the density can be expressed in terms of the quasi-particle amplitudes as, $\rho^N_{x\sigma} = \sum_s f^*_s(x\sigma)f_s(x\sigma)$, and that all the quasi-particle amplitudes with $s > 0$ decay exponentially faster than f_0, we can assume that the exponential tail of the density far from the surface of a finite system will be governed by the ground state amplitude f_0. The information on the ionization energy is therefore encoded in the decay of the density far from the bulk system's surface and it can be obtained in a DFT calculation. In particular, the relation between the ionization energy and the highest Kohn-Sham eigenvalue becomes clear if we consider the analogy between equation (1) and Kohn-Sham equations.We note indeed that, since the effective potential can be chosen to vanish in the vacuum far from the surface, in Kohn-Sham formalism the decay of the density is simply governed by the highest occupied Kohn-Sham level which thus coincides with the ionization energy.

The ionization energy as a function of U is shown in Fig. 1 : we see that Koopmans theorem of DFT is very well verified, the small error, within 2%, is due to the finite size of the vacuum chain.

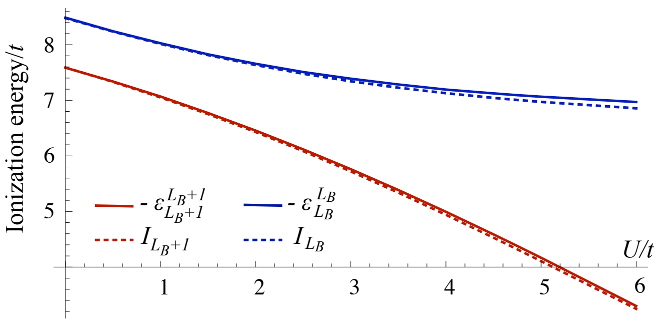

FIG. 1: **Numerical test of Koopmans theorem**. Ionization energy computed from the exact ground state energies compared with the highest occupied Kohn-Sham eigenvalue for the system with L_B and $L_B + 1$ particles as a function of U/t for constant bulk potential $w_0 = -8t$.

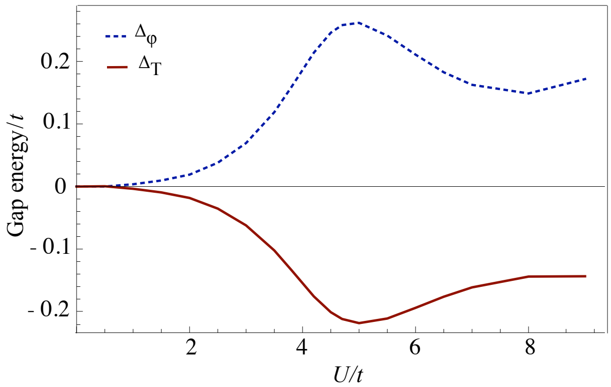

FIG. 2: **Finite-size corrections to the charge gap.** Δ_φ and Δ_T as functions of the ratio, U/t. The bulk potential is $v_x = 2(-1)^x$ while the nearest-neighbor interaction parmeter V equals zero. Similar results are obtained both for $V \neq 0$ and for $v_x = 0$.

Charge gap, Kohn-Sham gap and exchange correlation potential discontinuity

For sake of completeness we recall here the derivation of the relation between the charge-gap and the exchange-correlation potential shift starting from Koopmans theorem, this also will allow us to identify the corrections due to finite size effects.

By applying Koopmans theorem Δ_C can be rewritten as follows:

$$\Delta_C = \Delta_{KS} + \varepsilon_{N+1}^{N+1} - \varepsilon_{N+1}^{N} \tag{3}$$

where ε_N^M indicates the N-th Kohn-Sham level of the M-particle system and the gap in the Kohn-Sham spectrum of the N-particle system is given by $\Delta_{KS} = \varepsilon_{N+1}^N - \varepsilon_N^N$.

Using Kohn-Sham equations for the system with N and $N+1$ particles the charge gap can be further partitioned as the sum of four terms, namely

$$\Delta_C = \Delta_{KS} + \Delta_{xc} + \Delta_\varphi + \Delta_T \tag{4}$$

where Δ_{xc} is the exchange-correlation gap introduced in the article, essentially equivalent to the one used in the literature (see *e.g.* Ref. 4)

$$\Delta_{xc} = \sum_x |\varphi_{N+1}^{N+1}(x)|^2 \left(v_{\text{KS}}^{N+1}(x) - v_{\text{KS}}^N(x)\right) \tag{5}$$

while Δ_φ and Δ_T are due to the relaxation of the $N+1$-th Kohn-Sham orbital induced by the addition of a particle,

$$\Delta_T = \sum_x \left(\varphi_{N+1}^{N+1}(x)\hat{T}_s\varphi_{N+1}^{N+1}(x) - \varphi_{N+1}^{N}(x)\hat{T}_s\varphi_{N+1}^{N}(x)\right), \tag{6}$$

$$\Delta_\varphi = \sum_x \left(|\varphi_{N+1}^{N+1}(x)|^2 - |\varphi_{N+1}^{N}(x)|^2\right) v_{\text{KS}}^N(x). \tag{7}$$

where $v_{\text{KS}}^N(x)$ denotes the effective potential of the N-particle system.

For a translationally invariant system both Δ_φ and Δ_T become of order $1/N$ and can be neglected in the thermodynamic limit. Since the difference $\delta v_{\text{KS}}(x) = v_{\text{KS}}^{N+1}(x) - v_{\text{KS}}^N(x)$ becomes constant, in this limit, equation (4) reduces to the well-know relation between the charge gap and the exchange-correlation potential discontinuity shown *e.g.* in Ref. 3.

Interestingly we find that Δ_φ and Δ_T tend to cancel in finite systems in all interaction regimes. As an example in Figure 2 we plot the two contributions as a function of U/t for the ionic Hubbard model with $v_x = 2(-1)^x$ and $V = 0$, as one can see the two terms are finite but with opposite signs.

By applying perturbation theory it is not difficult to show that the cancellation stems directly from the definition of exchange-correlation gap given in Eq. (5). The effective potential, $v_{\rm KS}^{N+1}$, of the $N+1$ particle system can be indeed related to the potential, $v_{\rm KS}^{N}$, as follows

$$v_{\rm KS}^{N+1} = v_{\rm KS}^{N} + C + \Delta v \tag{8}$$

where C is a constant shift, $C = 1/(L_B + L_V)\sum_x \left(v_{\rm KS}^{N+1} - v_{\rm KS}^{N}\right)$, which accounts for the discontinuity and remains finite in the infinite size limit and Δv is a weak site dependent modulation such that $\sum_x \Delta v = 0$ which vanishes in the infinite size limit. Starting from Eq. (8), by applying perturbation theory in Δv we obtain the following expression for $\varepsilon_{N+1}^{N+1} - \varepsilon_{N+1}^{N}$:

$$\begin{aligned} \varepsilon_{N+1}^{N+1} - \varepsilon_{N+1}^{N} \;&\simeq\; C + \langle \varphi_{N+1}^{N}(x)|\Delta v|\varphi_{N+1}^{N}\rangle + \\ &+ \sum_{\nu\neq N+1} \frac{|\langle \varphi_{N+1}^{N}|\Delta v|\varphi_{\nu}^{N}\rangle|^2}{\varepsilon_{N+1}^{N} - \varepsilon_{\nu}^{N}} \end{aligned} \tag{9}$$

Similarly, by inserting Eq. (8) in Eq. (5) we can recast Δ_{xc} as

$$\Delta_{xc} = C + \langle \varphi_{N+1}^{N+1}|\Delta v|\varphi_{N+1}^{N+1}\rangle \tag{10}$$

Eventually, considering that $\Delta_\varphi + \Delta_T = \varepsilon_{N+1}^{N+1} - \varepsilon_{N+1}^{N} - \Delta_{xc}$ and expanding the wave-function $|\varphi_{N+1}^{N+1}\rangle$ in Eq. (10) we arrive at the following result:

$$\Delta_\varphi + \Delta_T \simeq -\sum_{\nu\neq N+1} \frac{|\langle \varphi_{N+1}^{N}|\Delta v|\varphi_{\nu}^{N}\rangle|^2}{\varepsilon_{N+1}^{N} - \varepsilon_{\nu}^{N}} \tag{11}$$

From this equation we see that the sum $\Delta_\varphi + \Delta_T$ is of second order in Δv, since the change in the wave-function is of first order in Δv.

With a similar reasoning one can prove that with the definition of Δ_{xc} given in Ref.4 the finite-size errors to second order in Δv have the same modulus but opposite sign.

---

[1] Almbladh C O and von Barth U 1985 *Physical Review B* **31** 3231
[2] Katriel J and Davidson E R 1980 *Proceedings of the National Academy of Sciences* **77** 4403
[3] Perdew J P and Levy M 1983 *Physical Review Letters* **51** 1884
[4] Sham L J and Schlüter M 1983 *Physical Review Letters* **51** 1888
